# Singletrack: an algorithm for improving memory consumption and performance of gap-affine sequence alignment

**DOI:** 10.1093/bioinformatics/btag183

**Published:** 2026-04-13

**Authors:** Lorién López-Villellas, Cristian Iñiguez, Albert Jiménez-Blanco, Quim Aguado-Puig, Miquel Moretó, Jesús Alastruey-Benedé, Pablo Ibáñez, Santiago Marco-Sola

**Affiliations:** Departamento de Informática e Ingeniería de Sistemas/Aragón Institute for Engineering Research (I3A), Universidad de Zaragoza, Zaragoza, 50018, Spain; Barcelona Supercomputing Center, Barcelona, 08034, Spain; Barcelona Supercomputing Center, Barcelona, 08034, Spain; Department of Computer Science, Universitat Politècnica de Catalunya, Barcelona, 08034, Spain; Departament d’Arquitectura de Computadors i Sistemes Operatius, Universitat Autònoma de Barcelona, Barcelona, 08193, Spain; Barcelona Supercomputing Center, Barcelona, 08034, Spain; Department of Computer Science, Universitat Politècnica de Catalunya, Barcelona, 08034, Spain; Departamento de Informática e Ingeniería de Sistemas/Aragón Institute for Engineering Research (I3A), Universidad de Zaragoza, Zaragoza, 50018, Spain; Departamento de Informática e Ingeniería de Sistemas/Aragón Institute for Engineering Research (I3A), Universidad de Zaragoza, Zaragoza, 50018, Spain; Barcelona Supercomputing Center, Barcelona, 08034, Spain; Department of Computer Science, Universitat Politècnica de Catalunya, Barcelona, 08034, Spain

## Abstract

**Motivation:**

Advances in DNA sequencing have outpaced advances in computation, making sequence alignment a major bottleneck in genome data analyses. Classical dynamic programming (DP) algorithms are particularly memory-intensive, especially when computing gap-affine and dual gap-affine alignments. Existing strategies to reduce memory consumption often sacrifice speed or alignment accuracy.

**Results:**

We present Singletrack, an efficient algorithm for backtrace gap-affine and dual gap-affine alignments that requires storing a single DP matrix while preserving optimal alignment results. Compared to classical DP algorithms, Singletrack removes the need to store additional matrices (i.e. 2 for gap-affine and 4 for dual gap-affine), significantly reducing memory consumption and, in turn, reducing pressure on the memory hierarchy and improving overall performance. Most importantly, Singletrack is a general backtrace method compatible with state-of-the-art DP-based algorithms and heuristics, such as the Suzuki-Kasahara (SK) and the Wavefront Alignment (WFA) algorithms. We demonstrate that Singletrack reduces memory consumption for both SK and WFA algorithms, lowering SK usage by 2× and 4× and WFA usage by 3× and 5× for gap-affine and dual gap-affine alignments, respectively. Moreover, replacing KSW2’s memory-reduction technique with Singletrack accelerates its SK implementation by up to 1.4× at the cost of doubling memory consumption, while Singletrack increases the performance of the WFA implementation in WFA2-lib by 1.2–2.1×. Compared to the efficient linear-memory BiWFA algorithm, the Singletrack-accelerated version of WFA trades a practical increase in memory usage for up to 5.2× higher performance.

**Availability and implementation:**

The Singletrack implementations presented in this work are available on Zenodo (DOI: 10.5281/zenodo.18770585) and GitHub (https://github.com/LorienLV/singletrack).

## 1 Introduction

The rapid advancements in DNA sequencing technologies over the past years have enabled the generation of vast amounts of sequencing data at unprecedented speeds (Goodwin et al. 2016). The massive surge in sequencing data production has introduced a computational bottleneck in large-scale sequence analyses (Pfeifer 2017, Alser et al. 2020, 2022). Notably, sequence alignment remains one of the most computationally demanding tasks in modern bioinformatics pipelines, such as read mapping (Schbath et al. 2012, Alser et al. 2021), de novo assembly (Jain et al. 2018, Liao et al. 2019), and pangenome construction (Wang et al. 2022, Garrison et al. 2024).

The goal of pairwise sequence alignment is to find a sequence of operations (i.e. match, mismatch, insertion, and deletion) that maps one sequence to another while minimizing a given cost function. In practice, the gap-affine and dual gap-affine cost functions are preferred due to their ability to model biologically accurate alignments.

Classical dynamic programming (DP) algorithms are commonly used to compute gap-affine and dual gap-affine alignments. In their basic formulation, these algorithms require computing and storing multiple DP matrices (i.e. 3 for gap-affine and 5 for dual gap-affine), each of size (n+1)×(m+1), where *n* and *m* are the input sequence lengths. These matrices are later traced back to recover the optimal alignment.

Building upon this classical formulation, several modern alignment algorithms have been proposed to improve execution time and reduce memory usage. One notable example is the Suzuki-Kasahara (SK) formulation (Suzuki and Kasahara 2018), which reformulates the gap-affine scoring equations so that the matrices store score differences instead of absolute scores. This formulation increases the number of matrices to store, but reduces the size of each cell, resulting in overall memory savings. This approach is used by KSW2 (Li 2018), at the core of Minimap2 (Li 2018), and by the libgaba (Suzuki and Kasahara 2018) library. More recently, the Wavefront Alignment Algorithm (WFA) (Marco-Sola et al. 2021) was proposed, which exploits homologous regions between sequences to substantially reduce the number of DP matrix cells computed and lower the memory requirements, particularly when aligning highly similar sequences.

Nevertheless, these implementations store all the computed cells of the DP matrices. In both the classical formulation and WFA, this involves storing three matrices for gap-affine and five for dual gap-affine alignment. In the case of the SK formulation, it requires storing four matrices for gap-affine alignment and six for dual gap-affine alignment. Compared to simpler cost functions, such as edit distance, using multiple matrices significantly increases memory requirements, making these algorithms particularly memory-intensive and degrading overall performance, especially when scaling to long sequences or when processing large alignment batches.

To address this challenge, practical sequence alignment implementations for gap-affine and dual gap-affine scoring adopt a variety of techniques to reduce memory usage. These include using heuristics at the expense of losing accuracy, storing compact backtrace representations instead of full matrices, and space-time trade-offs, such as divide-and-conquer strategies that recompute partial results to avoid storing the DP matrices entirely.

Heuristic-based pruning is a common technique to reduce memory usage by restricting the number of DP cells computed and stored. For instance, static banding restricts DP computations to a fixed diagonal band around the main diagonal, where the optimal alignment is expected to be found (Ukkonen 1985). Alternatively, the computation region can be adjusted dynamically during alignment, pruning cells that fall too far from the most promising paths (Altschul et al. 1997, Li 2016). Such heuristics may drastically reduce memory usage but risk missing the optimal alignment if it lies outside the explored region. Notwithstanding, they are standard (usually optional) features in many libraries, including KSW2 (Li 2018), Edlib (Šošić and Šikić 2017), Parasail (Daily 2016), and WFA2-lib (Marco-Sola et al. 2021).

Another memory-saving technique involves storing a backtrace matrix instead of the full DP matrices during alignment. That is, the aligner keeps only those DP cells required to compute subsequent DP cells, while the backtrace information (i.e. the direction of the next DP cell during the backtrace) is stored in a compact auxiliary matrix using small-width elements (e.g. 8-bit values). This approach is implemented in widely-used libraries such as KSW2 (Li 2018) and Parasail (Daily 2016). While effective at reducing memory consumption, this method introduces significant overhead from additional data manipulation and memory accesses during the alignment process.

Another common approach is to trade additional computation for reduced memory usage using a divide-and-conquer approach. The divide-and-conquer paradigm can be leveraged to recursively break the alignment process into smaller pieces, effectively transforming the quadratic memory consumption of classical algorithms to linear memory consumption. This technique is used by Hirschberg’s algorithm (Hirschberg 1975), FORAlign Wei et al. (2024), which accelerates Hirschberg by using the Four Russians method Arlazarov et al. (1970), and BiWFA (Marco-Sola et al. 2023), a version of WFA capable of exploiting divide-and-conquer. However, these linear-memory approaches, while extremely memory-efficient, tend to be slower than their quadratic-memory counterparts for moderate sequence lengths. For instance, the BiWFA paper reports that WFA outperforms BiWFA for sequences of up to 100 Kbp.

This work presents Singletrack, an efficient backtracing algorithm that requires storing only a single DP matrix, even when computing gap-affine and dual gap-affine alignments. We demonstrate that storing a single DP matrix is enough to recover the optimal alignment while maintaining both the linear time complexity of the backtrace and the optimality of the alignments. Moreover, we show that Singletrack is broadly applicable with state-of-the-art DP-based methods, such as SK formulation, WFA, and commonly used heuristic strategies. To demonstrate applicability and performance, we integrated Singletrack into KSW2 (which implements SK and uses the backtrace matrix approach to reduce memory consumption) and WFA2-lib (which implements WFA), yielding the KSW2+Singletrack and WFA+Singletrack optimized implementations.

We show that Singletrack significantly reduces memory consumption in both SK and WFA, thereby mitigating memory pressure and often translating into performance gains. In the SK formulation, memory consumption is reduced by 2× and 4× for gap-affine and dual gap-affine alignments, respectively. Although Singletrack increases memory usage by 2× compared to the memory reduction approach used in KSW2, it reduces execution time by up to 1.4×. In WFA, Singletrack lowers memory usage by 3× and 5× for gap-affine and dual gap-affine, respectively, and delivers speedups of 1.2× to 2.1× compared to the implementation in WFA2-lib. Compared to the linear-space BiWFA algorithm implemented in WFA2-lib, WFA+Singletrack achieves speedups of up to 5.2× at the cost of increased memory usage, making it a compelling alternative for moderately long sequences.

## 2 Background

### 2.1 Pairwise sequence alignment

Let the query $q=q_{0},q_{1},\ldots ,q_{n-1}$ and the target $t=t_{0},t_{1},\ldots ,t_{m-1}$ be two sequences of length *n* and *m* characters, respectively. The optimal sequence alignment is the sequence of four basic operations (i.e. match, mismatch, insertion, and deletion) that transform *q* into *t* while minimizing a given cost function.

Naturally, the choice of cost function strongly affects the alignment result and its biological relevance, as well as the computational and memory requirements. The simplest cost functions are the edit-distance and gap-linear (a.k.a. weighted edit-distance). These functions use three values to score matches (*a*), mismatches (*x*), and gap extensions (*e*) for both insertions and deletions. In the case of the edit-distance, these values are fixed to a=0, x=1, e=1, while gap-linear functions allow customizable values to match biological or empirical scoring needs. In the following, for simplicity, we define a substitution function S(i,j) that evaluates to *a* if $q_{i-1}=t_{j-1}$ and *x* if $q_{i-1}\neq t_{j-1}$.

Edit-distance and gap-linear alignments are traditionally computed using DP algorithms that require computing and storing a single DP matrix *M* of size (n+1)·(m+1), where each cell $M_{i,j}$ is filled using a simple recurrence, $M_{i,j}=\min\{M_{i,j-1}+e,M_{i-1,j}+e,M_{i-1,j-1}+S(i,j)\}$. As a result, the optimal score of the alignment is found in $M_{n,m}$, and the optimal alignment can be recovered by tracing back from this cell the sequence of minimum-cost operations that led to it, using the backtrace algorithm.

### 2.2 Gap-affine and dual gap-affine alignment

Despite their simplicity, gap-linear cost functions have notable limitations. These functions model long gaps as multiple independent insertions or deletions, whereas in reality, they often stem from a single biological event. This can lead to fragmented alignments and misleading interpretations, making linear models unsuitable in some cases. To reflect this phenomenon, the gap-affine function (Smith et al. 1981, Gotoh 1982) introduces a gap-opening cost ($o_{1}$). This function models cases where a gap follows a different type of operation, such as a mismatch or match. For example, two insertions separated by a non-insertion costs $2\cdot o_{1}+2\cdot e_{1}$, while a single gap of two insertions costs $o_{1}+2\cdot e_{1}$.

However, gaps in sequence alignments arise from two distinct mechanisms: evolutionary events, which can introduce long gaps through a single event (e.g. transposon insertion), and sequencing errors, which tend to generate multiple short gaps. To model this distinction and produce more biologically accurate alignments, the dual gap-affine function (Gotoh 1990) is often preferred. To that end, the dual gap-affine function introduces a second set of gap penalties ($o_{2},e_{2}$), allowing for two types of gaps. One type of gap has a low opening cost but a high extension cost (to model short gaps), while the other has a high opening cost but a low extension cost (to model large gaps). For each gap of length *l*, the alignment algorithm chooses the lower-cost option: $\min\{o_{1}+l\cdot e_{1},o_{2}+l\cdot e_{2}\}$.

Similar to gap-linear models, gap-affine and dual gap-affine alignments are typically computed using traditional DP algorithms. However, computing gap-affine alignments requires computing and storing three DP matrices (*M*, *I*1, and *D*1). More demanding still, dual gap-affine alignment requires five matrices (*M*, *I*1, *D*1, *I*2, and *D*2). In particular, the *I* matrices (*I*1 and *I*2) and *D* matrices (*D*1 and *D*2) track optimal scores for alignments ending in insertions and deletions, respectively. Then, the *M* matrix summarizes the minimum alignment score between prefixes $q_{0,\ldots ,i-1}$ and $t_{0,\ldots ,j-1}$ for each cell $M_{i,j}$. The recurrence equation used to compute the gap-affine DP matrices is presented in Equation (1). Additional details, including initial conditions and the extension to the dual gap-affine cost function, are provided in the Supplementary Material, available as supplementary data at *Bioinformatics* online.


(1) $$\begin{array}{c} I1_{i,j}=\min\{I1_{i,j-1}+e_{1},M_{i,j-1}+o_{1}+e_{1}\}\\ D1_{i,j}=\min\{D1_{i-1,j}+e_{1},M_{i-1,j}+o_{1}+e_{1}\}\\ M_{i,j}=\min\{I1_{i,j},D1_{i,j},M_{i-1,j-1}+S(i,j)\} \end{array}$$

As in gap-linear models, the optimal alignment score is located in cell $M_{n,m}$. To reconstruct the alignment, a backtrace procedure traverses the *M*, *I*, and *D* matrices, following the path of minimum-cost operations that led to this cell (i.e. optimal alignment).

### 2.3 Classical backtrace algorithm

The classical backtrace algorithm reconstructs an optimal alignment path by traversing the *M*, *I*, and *D* matrices. More in detail, the backtrace procedure starts at the last cell, $M_{n,m}$. At each step, the algorithm identifies the predecessor cell that originated the score of the current cell by evaluating the transitions defined in Equation (1). This transition corresponds to an alignment operation, belonging to the optimal alignment, which is commonly represented using the CIGAR string format: M for match, X for mismatch, I for insertion, and D for deletion. The algorithm then moves to the identified predecessor cell and repeats the process until it reaches the starting cell $M_{0,0}$. It is important to note that multiple transitions may lead to the same minimum-cost cell, resulting in different equally optimal alignment paths. In the case of the dual gap-affine function, the backtrace algorithm is analogous.

**Figure 1:**
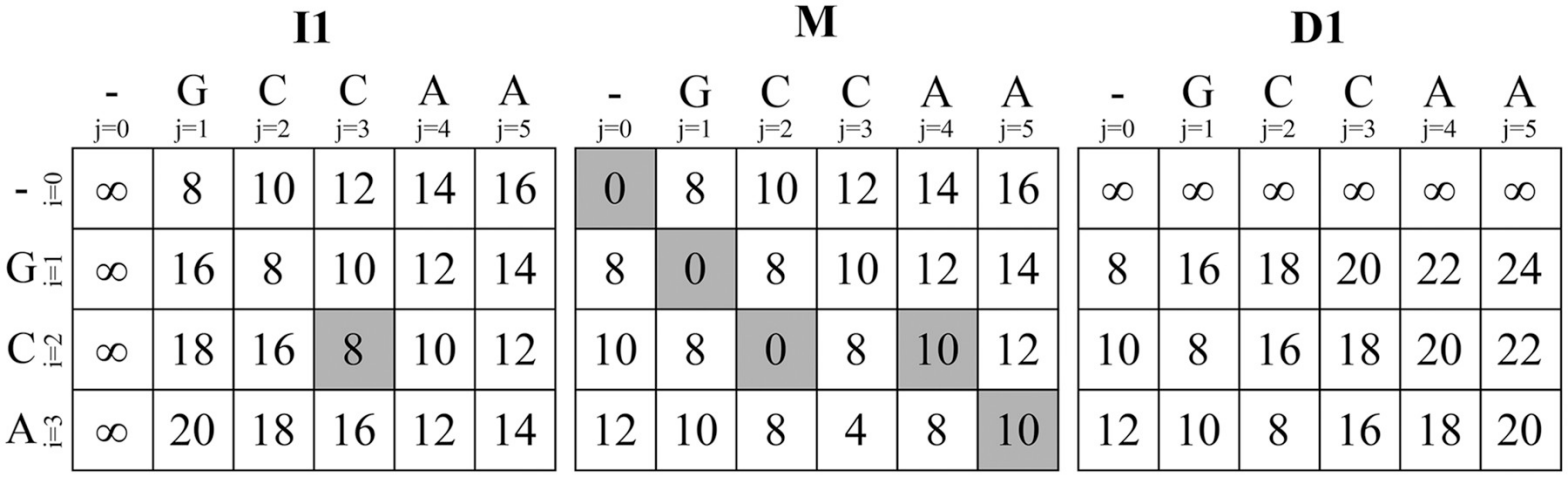
Classical backtrace for the gap-affine alignment of q=GCA and t=GCCAA using penalties a=0, x=4, $o_{1}=6$, and $e_{1}=2$. Dark-shaded cells indicate the optimal path over matrices *M*, *I*1, and *D*1.

For example, Figure 1 shows how the classical backtrace algorithm operates over the computed DP matrices when aligning two sequences (q=GCA and t=GCCAA) using a gap-affine cost function with penalties a=0, x=4, $o_{1}=6$, and $e_{1}=2$. The dark-shaded cells in the figure indicate the optimal alignment path. The backtrace begins at cell M[n,m]=M[3,5]=10. The only valid transition is from M[2,4], since M[3,5]=M[2,4]+S(3,5). The current cell is updated to M[2,4], and the CIGAR string is set to M. From M[2,4], the algorithm moves to I1[2,3], as $M[2,4]=I1[2,3]+e_{1}$, updating the CIGAR string to IM. Then, since $I1[2,3]=M[2,2]+o_{1}+e_{1}$, the path returns to M[2,2], resulting in IIM. Next, M[2,2] traces back to M[1,1] via M[2,2]=M[1,1]+S(2,2), giving MIIM. Finally, M[1,1]=M[0,0]+S(1,1), and the completed CIGAR string is MMIIM. This example illustrates how the presence of a gap in the alignment requires transitioning into *I*1 or *D*1 during backtrace and returning to *M* once the gap is closed.

## 3 The singletrack algorithm

We now describe Singletrack, a method for performing the backtrace of gap-affine and dual gap-affine alignments in linear execution time using only the *M* matrix. As a result, the alignment process no longer needs to store the indel matrices, significantly reducing memory usage., For simplicity, we focus on the gap-affine case, noting that the method extends naturally to dual gap-affine alignments. The pseudocode for the Singletrack algorithm applied to the dual gap-affine case is provided in the Supplementary Material, available as supplementary data at *Bioinformatics* online.

We observe that Equation (1) shows that while computing *M* requires values from all three matrices, each indel matrix (*I*1, *D*1) depends only on *M* and itself. Thus, any cell in an indel matrix traces back either to a cell in *M* or to another cell within the same indel matrix. Hence, during backtrace, if the current cell is in an indel matrix such as *I*1 with score *s*, we can avoid accessing *I*1 by checking first whether $s=M_{i,j-1}-o_{1}-e_{1}$. If this holds, the gap was opened from $M_{i,j-1}$; otherwise, the gap continues, and the predecessor is necessarily $I1_{i,j-1}$ with score $s^{\prime }=s-e_{1}$. As a result, backtracing through *I*1 can be performed iteratively without accessing its scores, until the trace returns to a cell in *M*. Singletrack generalizes this idea to avoid accessing *I*1 and *D*1 scores. When the current cell is in *M*, say $M_{i,j}$, we first check if the predecessor is in *M* by checking if $M_{i,j}=M_{i-1,j-1}+S(i,j)$. If not, the predecessor cell is either $I1_{i,j-1}$ or $D1_{i-1,j}$. Although we do not know which one, from Equation (1), we know that the score of the predecessor cell must be $s^{\prime }=M_{i,j}-e_{1}$. At this point, we explore two tentative backtrace paths simultaneously: one assuming the predecessor lies in *I*1, and the other in *D*1. Then, we extend both paths step by step, increasing the gap length *l* and computing the expected score to return to *M* as $s(l)=M_{i,j}-o_{1}-l\cdot e_{1}$. Eventually, either $M_{i,j-l}=s(l)$ or $M_{i-l,j}=s(l)$ holds, indicating that the correct backtrace path goes from $M_{i,j}$ to $M_{i,j-l}$ through *I*1 (insertion) or to $M_{i-l,j}$ through *D*1 (deletion), respectively. Once a consistent path back to *M* is found, the other path is discarded, and the algorithm continues the backtrace in *M*. The pseudocode of Singletrack’s backtrace algorithm is shown in Algorithm 1. Note that Singletrack always finds an optimal path, just as the classical backtrace algorithm does. If multiple optimal paths exist, it will select any of them, ensuring optimality in all cases.

Algorithm 1: Singletrack gap-affine backtrace

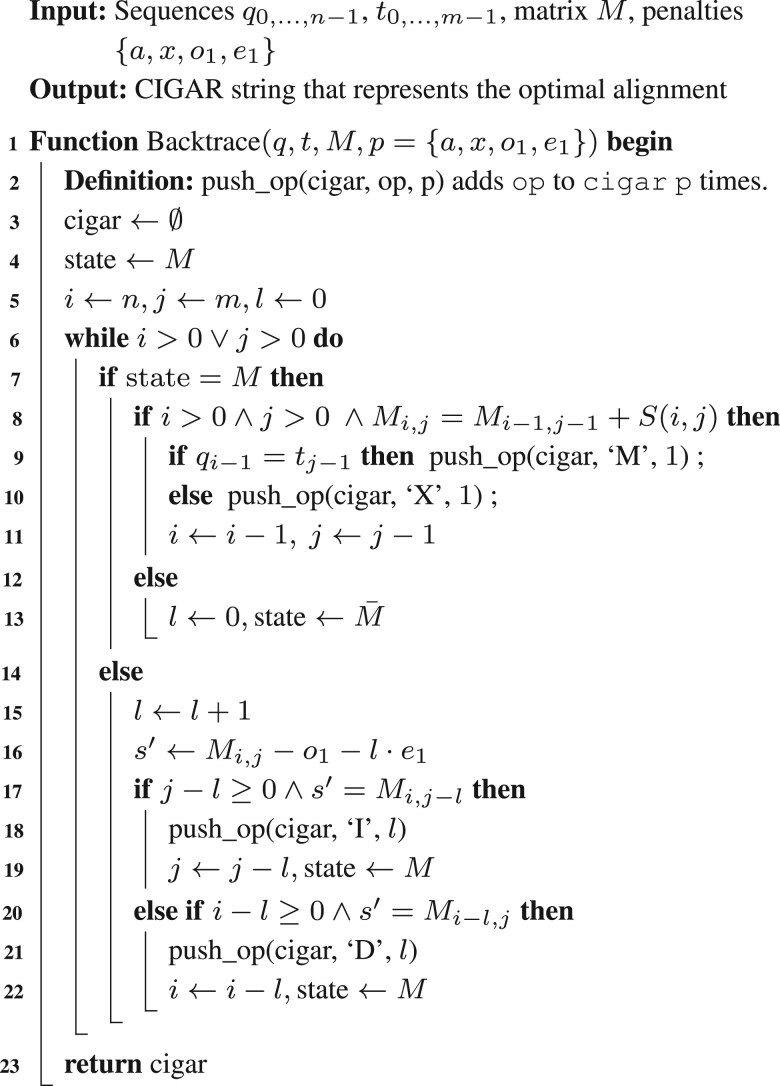

**Figure 2:**
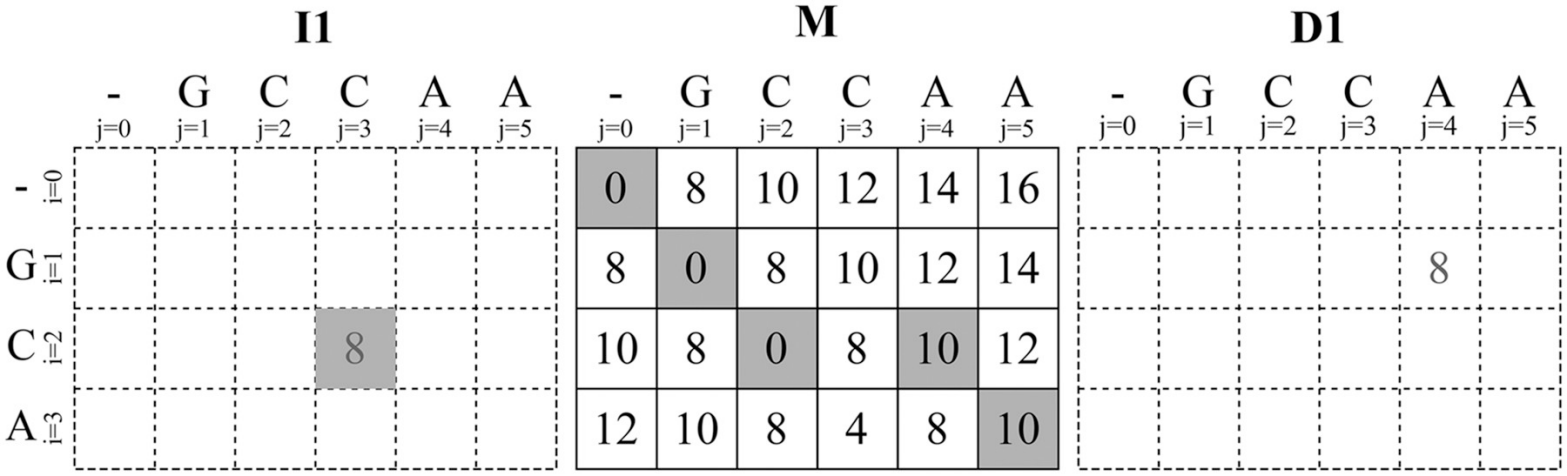
Singletrack backtrace for the gap-affine alignment of q=GCA and t=GCCAA using penalties a=0, x=4, $o_{1}=6$, and $e_{1}=2$. Only the matrix *M* is stored. Strictly required *I*1 and *D*1 values are recomputed on the fly. Dark-shaded cells indicate the optimal path.

Following the previous example, Figure 2 shows the behaviour of the Singletrack backtrace algorithm. For that, we show both the stored and recomputed cells: scores stored in memory (corresponding to the *M* matrix) are displayed in black, while tentative scores, computed on the fly during the backtrace but not stored, are shown in dark grey. In the first step, the behaviour matches the classical algorithm since $M_{3,5}=M_{2,4}+S(3,5)$ and the CIGAR string is set to M. At $M_{2,4}$, since $M_{2,4}\neq M_{1,3}+S(2,4)$, the predecessor cell must be either $I1_{2,4}$ or $D1_{2,4}$, both having a tentative score $s^{\prime }=M_{2,4}=10$. For a tentative gap length of l=1, the expected score back to *M* should be $s^{\prime }=M_{2,4}-o_{1}-l\cdot e_{1}=2$, which does not correspond to $M_{2,3}$ or $M_{1,4}$. Note that the tentative score of $I1_{2,3}$ and $D1_{1,4}$ is $s^{\prime }=M_{2,4}-e_{1}=8$, corresponding to the true score at $I1_{2,3}$ (correct backtrace path) but not of $D1_{1,4}$ (see Fig. 1). Increasing the gap length to l=2, the algorithm extends both paths and checks whether the updated score back to *M*, $s^{\prime }=10-6-2\cdot 2=0$, matches either $M_{2,2}$ or $M_{1,3}$. Since $s^{\prime }=M_{2,2}$, the correct path goes through *I*1, and two insertions are added to the CIGAR string, which becomes IIM. From this point, the algorithm proceeds as in the classical case, and the final CIGAR string is MMIIM, matching the result produced by the original backtrace algorithm.

The time complexity of the Singletrack algorithm is linear O(n+m), the same as the classical backtrace. In the worst-case scenario, Singletrack may explore up to twice as many cells as the classical backtrace algorithm. However, the backtrace step generally represents only a small fraction of the total alignment time, which is dominated by the quadratic O(nm) time complexity of the alignment step. In the case of dual gap-affine alignments, the approach is identical, but with four tentative paths corresponding to *I*1, *D*1, *I*2, and *D*2, potentially increasing the worst-case number of explored cells by up to four times compared to the classical algorithm.

The key advantage of Singletrack lies in its ability to compute optimal alignments while storing only the *M* matrix and a minimal set of values from the other matrices (i.e. *I*1, *D*1, *I*2, and *D*2). In particular, to compute the DP cells using Equation (1) column-wise, the horizontal recurrences of *M* and *I*1 depend on the left neighbour $I1_{i,j-1}$, which requires storing the full previous column of *I*1. In contrast, the vertical recurrences of *M* and *D*1 depend on the top neighbour $D1_{i-1,j}$. Under column-wise iteration, this value corresponds to a single scalar that can be carried forward, so only one cell from *D*1 needs to be stored. Exploiting this property allows the alignment to be computed without any additional overhead while reducing memory usage by approximately 3× for gap-affine alignments. The same reasoning extends naturally to the dual gap-affine case by additionally storing a full column of *I*2 and a single cell of *D*2, resulting in a 5× reduction in memory consumption.

Moreover, requiring only a small set of the indel matrices enables software optimizations. For instance, the single element needed from *D*1 (and *D*2) can be stored directly in a CPU register rather than in memory, significantly reducing memory accesses and latency, and improving performance. Similarly, the memory of the single column required from *I*1 (and *I*2) can be reused continuously, which improves the cache locality and reduces pressure on lower levels of the memory hierarchy, which is particularly advantageous when aligning long sequences or processing large alignment batches.

More importantly, Singletrack can be applied to any DP-based sequence alignment algorithm, provided that the required scores of the *M* matrix are available during the backtrace. As shown in the following sections, Singletrack integrates seamlessly with the Suzuki-Kasahara formulation (Suzuki and Kasahara 2018) and with the Wavefront Alignment Algorithm (Marco-Sola et al. 2021). Additionally, it is compatible with heuristic alignment approaches, such as bands, early termination, drops, or score filtering, requiring only additional checks for boundary conditions during the backtrace. For simplicity, we focus on global alignment in this work, noting that the same idea extends naturally to semi-global, ends-free, and local alignments.

We have integrated the Singletrack backtrace into the classical dynamic programming algorithm. The implementation is available on Zenodo (DOI: 10.5281/zenodo.18770585) and GitHub (https://github.com/LorienLV/singletrack).

## 4 Singletrack in Suzuki-Kasahara formulation

The Suzuki–Kasahara (SK) formulation (Suzuki and Kasahara 2018) extends the difference encoding idea from the BPM algorithm (Myers 1999) to the gap-affine cost function, storing only the differences between adjacent DP cells rather than full scores. Since these differences and alignment penalties are usually small integers, they can be efficiently represented with reduced data types, such as 8-bit integers. The standard recurrence relations [Equation (1)] are reformulated in terms of score differences for the *M*, *I*1, and *D*1 matrices. Specifically, we define the horizontal, vertical, and diagonal differences in the *M* matrix as $\Delta H_{i,j}=M_{i,j}-M_{i,j-1}$, $\Delta V_{i,j}=M_{i,j}-M_{i-1,j}$, and $A_{i,j}=M_{i,j}-M_{i-1,j-1}$, respectively. Additionally, the differences between the *I*1 and *D*1 matrices and the *M* matrix are given by $\Delta E1_{i,j}=I1_{i,j}-M_{i,j}$ and $\Delta F1_{i,j}=D1_{i,j}-M_{i,j}$. The resulting Suzuki-Kasahara recurrence relations are shown in Equation (2) (see Supplementary Material, available as supplementary data at *Bioinformatics* online, for the dual gap-affine equations).


(2) $$\begin{array}{c} A_{i,j}=\min\{\begin{array}{c} S(i,j)\\ \Delta E1_{i,j-1}+\Delta V_{i,j-1}+e_{1}\\ \Delta F1_{i-1,j}+\Delta H_{i-1,j}+e_{1} \end{array}\\ \Delta H_{i,j}=A_{i,j}-\Delta V_{i,j-1}\\ \Delta V_{i,j}=A_{i,j}-\Delta H_{i-1,j}\\ \Delta E1_{i,j}=\min\{o_{1},\Delta E1_{i,j-1}-\Delta H_{i,j}+e_{1}\}\\ \Delta F1_{i,j}=\min\{o_{1},\Delta F1_{i-1,j}-\Delta V_{i,j}+e_{1}\} \end{array}$$

With these new recurrence relations, four matrices are stored: $\Delta H_{i,j}$, $\Delta V_{i,j}$, $\Delta E1_{i,j}$, and $\Delta F1_{i-1,j}$, compared to only three in the original formulation (where $A_{i,j}$ is a temporary variable and not stored in memory). However, since the new matrices store narrow-integer values (e.g. 8-bit integers), the overall memory footprint is actually reduced compared to the original approach (assuming 32-bit integer encoding baseline). Furthermore, this reformulation increases the number of elements that can be processed simultaneously through SIMD (Single Instruction, Multiple Data) operations available in modern CPUs, enhancing computational performance.

To apply the Singletrack backtrace under the SK formulation, it is sufficient to retain only the ΔV and ΔH matrices, as they provide all the information required to reconstruct the original *M* matrix (the remaining $\Delta E_{1}$ and $\Delta F_{1}$ matrices can be discarded). However, the Singletrack algorithm requires integer values from the *M* matrix to trace back the optimal alignment from $M_{n,m}$ to $M_{0,0}$. To address this, we define a new matrix $M^{\prime }$ such that $M_{i,j}^{\prime }=M_{i,j}-M_{n,m}$ for all i∈{0,…,n} and j∈{0,…,m}. By construction, $M_{n,m}^{\prime }=0$, providing a known integer value to start the backtrace. During the backtrace, the relevant entries of $M^{\prime }$ are reconstructed on the fly using the stored differential matrices ΔV and ΔH as $M_{i,j}^{\prime }=M_{i-1,j-1}^{\prime }+\Delta H_{i-1,j}+\Delta V_{i,j}$. Since $M^{\prime }$ is simply a shifted version of *M*, the original alignment path is preserved, and the general Singletrack algorithm remains unchanged. These ideas naturally extend to the SK formulation of the dual gap-affine cost function.

We have integrated Singletrack into KSW2 (https://github.com/lh3/ksw2), which provides a SIMD-accelerated implementation of the Suzuki-Kasahara formulation and serves as a core component of Minimap2 (Li 2018), a widely adopted read mapper and a foundational tool in many bioinformatics pipelines. KSW2 implements the previously described backtrace matrix approach to reduce memory consumption, in which the backtrace information for all DP cells is generated on the fly during alignment and stored in a single compressed matrix of 8-bit elements. This approach requires 4× and 6× less memory than the original Suzuki-Kasahara implementation for gap-affine and dual gap-affine, respectively. However, it introduces additional computation in the alignment step by forcing the precomputation of backtrace information for all DP cells, including those that are not accessed during traceback because they do not lie on the optimal alignment path.

We produced a minimal version of KSW2 that utilizes Singletrack for backtrace, referred to as KSW2+Singletrack. Instead of computing the backtrace matrix, the matrices ΔH and ΔV are stored. This approach requires approximately twice the memory compared to the original KSW2; however, it eliminates all data manipulation associated with the backtrace matrix. Note that KSW2+Singletrack uses 2× and 4× less memory than the classical approach that stores all matrices for gap-affine and dual gap-affine, respectively. The KSW2+Singletrack implementation is available at https://github.com/LorienLV/singletrack.

## 5 Singletrack in wavefront alignment algorithm

The Wavefront Alignment Algorithm (Marco-Sola et al. 2021) (WFA) proposes the alternative recurrence relations to compute the optimal gap-affine alignment. For each matrix (*M*, *I*1, *D*1) and diagonal *k* of the DP matrix, WFA computes and stores the offset (column) of the most advanced DP cell with score *z*, known as the furthest reaching point (f.r.p.). The vector of offsets for a given score *z* is called the *z*-wavefront. In the gap-affine case, WFA computes three wavefronts (${\tilde{M}}_{z}$, ${\tilde{I1}}_{z}$, ${\tilde{D1}}_{z}$) per each score value following Equation (3).


(3) $$\begin{array}{c} {\tilde{I1}}_{z,k}=\max\{{\tilde{M}}_{z-o_{1}-e_{1},k-1},{\tilde{I1}}_{z-e_{1},k-1}\}+1\\ {\tilde{D1}}_{z,k}=\max\{{\tilde{M}}_{z-o_{1}-e_{1},k+1},{\tilde{D1}}_{z-e_{1},k+1}\}\\ {\tilde{M}}_{z,k}=\max\{{\tilde{M}}_{z-x,k}+1,{\tilde{I1}}_{z,k},{\tilde{D1}}_{z,k}\} \end{array}$$

Once the wavefronts for the optimal score *s* have been computed, the alignment path is recovered through a backtrace procedure that follows the same principles as the classical backtrace algorithm. That is, the WFA backtrace reconstructs the optimal alignment path following the origin of each furthest-reaching point in Equation (3) from ${\tilde{M}}_{s}$ to ${\tilde{M}}_{0}$, accessing all computed wavefronts in between (i.e. ${\tilde{M}}_{z}$, ${\tilde{I1}}_{z}$, and ${\tilde{D1}}_{z}$)

Unlike classical DP-based alignment algorithms, which require quadratic time and memory, WFA has a time complexity of O(ns) and a space complexity of $O(s^{2})$, where *n* is the length of the sequences (assuming n∼m) and *s* is the optimal alignment score. In practice, WFA outperforms most sequence alignment algorithms, especially when the sequences aligned are highly similar.

As with the other methods, the Singletrack backtrace algorithm can be applied to WFA, requiring only to store the $\tilde{M}$ wavefronts. This process is analogous to that used in the other cases. In essence, the Singletrack algorithm follows the transitions in $\tilde{M}$ when the predecessor of the current cell is in $\tilde{M}$, and opens tentative paths in $\tilde{I1}$ and $\tilde{D1}$ when the predecessor is not in $\tilde{M}$ (see an extended description in the Supplementary Material, available as supplementary data at *Bioinformatics* online). Consequently, it is not necessary to store the $\tilde{I1}$ and $\tilde{D1}$ wavefronts. As in the previous sections, these ideas naturally extend to the dual gap-affine WFA.

We have integrated Singletrack into WFA2-lib (https://github.com/smarco/WFA2-lib), which implements the Wavefront Alignment Algorithm and is utilized in several state-of-the-art genome analysis tools, including Anchorwave (Song et al. 2022) and wfmash (Garrison et al. 2024). WFA2-lib explicitly stores all the wavefronts to perform the backtrace (i.e. for each score *z*, three wavefronts in the case of gap-affine alignment and five wavefronts in the case of dual gap-affine alignment).

In our version of WFA2-lib, WFA+Singletrack, only the $\tilde{M}$ wavefronts are stored and used for the backtrace. For the indel matrices, we allocate a minimal scope of *N* wavefronts at the start of the alignment, each wavefront with the maximum possible size that may be required during the alignment. These *N* wavefronts are initially assigned to scores z=0,…,N−1. As the alignment progresses and a wavefront’s score moves out of scope, its memory is recycled for higher scores; i.e. the wavefronts within the scope are cycled, ensuring that no additional memory is required for the indel matrices. For sufficiently large sequences, WFA+Singletrack reduces memory usage by a factor of 3× for gap-affine and 5× for dual gap-affine alignments compared to the original WFA implementation in WFA2-lib. The modified version of WFA2-lib supporting Singletrack is available at https://github.com/LorienLV/singletrack.

## 6 Results

### 6.1 Experimental setup

We evaluate the performance and memory consumption of KSW2+Singletrack and WFA+Singletrack, introduced in previous sections, and compare them with their original implementations. Additionally, we evaluate the divide-and-conquer version of WFA (BiWFA) as a state-of-the-art reference alignment algorithm with linear memory complexity. The classical dynamic programming implementation (with and without Singletrack) is omitted from the results due to its substantially higher computational cost compared to the optimized KSW2 and WFA algorithms. All algorithms are executed without heuristics and therefore always produce optimal alignments.

For the evaluation, we configure all implementations to compute global alignments using the default penalties of BWA-MEM Li (2013) and WFA2-lib. When computing gap-affine alignments, penalty values are set to a=0, x=4, $o_{1}=6$, and $e_{1}=2$. For dual gap-affine alignments, penalties are set to a=0, x=4, $o_{1}=6$, $e_{1}=2$, $o_{2}=24$, and $e_{2}=1$.

It is important to note that the chosen penalties do not affect the alignment time of KSW2 and KSW2+Singletrack, since the full DP matrices are computed in both cases. While the penalties can slightly increase traceback time, this overhead is negligible for both algorithms. In contrast, the chosen penalty values affect the execution time and memory usage of the WFA and BiWFA algorithms and, consequently, WFA+Singletrack. However, the relative increase in the number of computed cells, and therefore in memory consumption and execution time, remains the same across all these algorithms.

We evaluate our method on three datasets obtained from real sequencing experiments. For each dataset, the error rate is defined as the proportion of edit operations required to optimally align the query sequence into the target sequence. For example, an error rate of 15% for a query and target of length 100 indicates that 15 edit operations (i.e. mismatches, insertions, and deletions) are required.

The first, Illumina WGS, was obtained from NIST’s Genome in a Bottle (GIAB) project (https://github.com/genome-in-a-bottle/giab_data_indexes) and comprises 100 million sequence pairs with lengths ranging from 140 bp to 280 bp (average 248.38 bp) and an average error rate of 1%. The second, PacBio HiFi, was obtained from the Precision FDA Truth Challenge V2 (https://precision.fda.gov/challenges/10/intro) and contains 10 million sequence pairs ranging from 0.20 Kbp to 24.64 Kbp (average 12.87 Kbp) and an average error rate of 0.6%. The third dataset, ONT PromethION, was obtained from the Human Pangenome Reference Consortium (Miga and Wang 2021) and comprises 1312 sequence pairs with lengths of up to 309.28 Kbp (average 173.27 Kbp). For our experiments, we select only the pairs up to 50 Kbp in length, resulting in 18 sequences with an average length of 27.60 Kbp and an average error rate of 12%. While the full ONT dataset was preliminarily tested, only BiWFA could complete the alignments, as the other algorithms exceeded the compute node’s memory capacity. Consequently, our evaluation focuses on the subset of sequence pairs that can be aligned by all methods under comparison, ensuring a fair and informative comparison. It is important to note that aligning such long sequences (i.e. > 50 Kbp) is relatively uncommon in practice, as most mappers, such as Minimap2 (Li 2018), typically use a seed-and-extend approach that requires aligning much smaller sequence chunks (e.g. anchors or seeds) rather than full-length sequences. The curated datasets are available in Zenodo (DOI: 10.5281/zenodo.17525720).

All the experiments were conducted on a computing node equipped with two Intel Xeon Platinum 8480+ processors, each featuring 56 cores and 112 threads, and 256 GiB of main memory. The main specifications of the node are summarised in Table 1.

**Table 1:** Main features of the computing nodes used to execute the experiments.

Processor	2 × Intel Xeon Platinum 8480+
Cores	2 × 56
Frequency	2 GHz
L1 cache (I + D)	32 KiB + 48 KiB (per core)
L2 cache	2 MiB (per core)
LLC	2 × 105 MiB (shared)
Main Memory	256 GiB DDR5 (16×16 GiB@4800 MHz)

### 6.2 Single-threaded results

In this section, we evaluate the single-threaded performance of the original KSW2, WFA, and BiWFA implementations, compared with the Singletrack-improved variants introduced in this manuscript, KSW2+Singletrack and WFA+Singletrack, in terms of time and memory usage. Performance is evaluated by measuring the total execution time required to align complete datasets (including the backtrace phase), while memory consumption is measured as the peak resident set size (RSS) recorded during the alignment process using the GNU/usr/bin/time command. Table 2 summarizes the execution times and memory footprints for all five implementations using both gap-affine (aff) and dual gap-affine (2aff) cost functions. To enable comparison across datasets of varying sizes, the alignment times are normalized by the number of base pairs. For each dataset and cost function, the best-performing algorithm for each metric (performance or memory) is highlighted in bold.

**Table 2:** Single-threaded runtime and memory usage of KSW2, KSW2+ST, WFA, BiWFA, and WFA+ST (aff = gap-affine; 2aff = dual gap-affine; ST = Singletrack).

		Time (μs)/Kbp			Mem (MiB)		
		Illumina	PacBio HF	PromethION	Illumina	PacBio HF	PromethION
aff	KSW2	65.8	2,980.6	7,849.8	3.8	10,463.6	4,534.5
	KSW2+ST	60.8	3,634.5	10,063.8	4.0	21,864.9	9,065.2
	WFA	4.8	13.3	7,447.2	5.3	427.9	6,337.0
	BiWFA	8.9	18.6	**4,428.1**	**3.2**	**15.1**	**17.3**
	WFA+ST	**3.4**	**10.9**	5,233.2	26.0	179.5	2,123.8
2aff	KSW2	110.5	4,887.9	12,479.2	3.8	10,463.6	4,537.9
	KSW2+ST	77.7	4,108.7	**11,472.8**	4.0	21,864.9	9,066.2
	WFA	11.3	50.9	44,884.7	6.8	2,557.0	37,653.1
	BiWFA	28.5	172.8	35,223.5	**6.0**	**58.8**	**115.4**
	WFA+ST	**5.4**	**39.7**	28,782.6	62.4	543.8	7,546.1

The best-performing algorithm for each metric (performance or memory) is highlighted in bold.

KSW2+Singletrack requires twice the memory of the original KSW2 for both gap-affine and dual gap-affine implementations, as it stores the ΔH and ΔV matrices instead of the backtrace matrix used in KSW. However, KSW2+Singletrack eliminates all computations and memory accesses related to constructing this backtrace matrix. This change leads to performance improvements on the Illumina dataset when using the gap-affine cost function. When aligning short sequences, both implementations have a similar memory footprint, as the total memory usage is dominated by fixed overheads such as auxiliary structures and allocation thresholds. In contrast, aligning the PacBio HF and Promethion datasets results in a slowdown of 1.2× on average due to increased memory pressure, highlighting the critical importance of memory efficiency when scaling to long sequences. However, when computing dual gap-affine (2aff), which is the primary alignment mode in Minimap2 (Li 2018), the memory requirements for KSW2+Singletrack remain the same as in the case of gap-affine (aff). For the baseline KSW2, constructing the backtrace matrix requires more instructions due to the additional matrices involved in dual gap-affine. As a result, KSW2+Singletrack achieves consistent speedups on all three datasets, ranging from 1.1× to 1.4×, while presenting a simpler implementation and minimal integration overhead.

Among all the studied algorithms, KSW+Singletrack outperforms KSW2 in four of the six experiments in terms of execution time, consuming roughly twice as much memory in the runs with the PacBioHF and PromethION datasets. Furthermore, KSW+Singletrack demonstrates performance advantages over BiWFA, WFA, and WFA+Singletrack on the PromethION dataset under dual gap-affine const function, while also exhibiting lower memory usage than WFA in that same experiment.

Regarding WFA+Singletrack, it outperforms the original WFA, achieving 1.2–2.1× speedups and reaching the theoretical 3× and 5× memory reductions for gap-affine (aff) and dual gap-affine (2aff), respectively, on the dataset with the longest sequences (PromethION). It should be emphasized that WFA+Singletrack does not use the same amount of memory in gap-affine and dual gap-affine, since in the latter it explores a larger number of cells in the dynamic programming (DP) matrix, i.e. the $\tilde{M}$ wavefronts require more memory.

In terms of execution time improvement, note that WFA+Singletrack repeatedly reuses the memory allocated to the indel matrices, reducing cache misses compared to WFA, especially for larger sequences. By examining the memory usage reported in the tables, it is clear that the alignment data exceeds the capacity of both the L1D cache (48 KiB) and the L2 cache (2 MiB) across all datasets and algorithm variants. Therefore, reducing cache misses has a significant positive impact on performance.

In terms of memory consumption, WFA+Singletrack uses less memory than WFA, except for the Illumina dataset. The difference in memory usage increases as the sequence length grows, eventually reaching theoretical maximum reductions of 3× and 5×. For Illumina reads, WFA+Singletrack over-allocates memory for indel matrices assuming worst-case usage, while most of it remains unused. Despite this, it outperforms WFA 1.3× for gap-affine and 2.1× for dual gap-affine, justifying the trade-off given the low absolute memory cost.

The linear-memory BiWFA algorithm also benefits from efficient memory reuse, leading to fewer cache misses than the original WFA. However, BiWFA’s divide-and-conquer approach introduces additional overhead by recursively recomputing already-processed alignment regions. This overhead translates to WFA+Singletrack outperforming BiWFA on the Illumina and PacBio HiFi datasets for gap-affine and in all three datasets for dual gap-affine. Across the reported results, WFA+Singletrack is up to 5.2× faster than BiWFA. Although BiWFA consistently exhibits the lowest memory consumption across all datasets, the substantial performance improvements offered by WFA+Singletrack make it a compelling choice for moderately long sequences, where memory usage remains manageable.

Among all the studied algorithms, WFA+Singletrack demonstrates better performance and memory efficiency than WFA in all experiments. It also outperforms BiWFA in four of the six experiments, though at the cost of higher memory usage. Compared to KSW2 and KSW2+Singletrack, WFA+Singletrack delivers better performance in five of the six experiments, only being outperformed by KSW+Singletrack on the PromethION dataset under gap-affine, while still using less memory.

Overall, in five of the six experiments, the top-performing algorithm is one that uses Singletrack (either KSW2+Singletrack or WFA+Singletrack). Notably, Singletrack consistently improves the memory efficiency of WFA, while in KSW2 it strikes a balance between the aggressive backtrace matrix approach of KSW2 and the original Suzuki-Kasahara implementation, providing performance benefits for short to moderately large sequences.

### 6.3 Multi-threaded results

Modern sequence alignment tools are frequently deployed in multi-threaded settings to take advantage of today’s multi-core architectures. This is especially true in large-scale applications such as read mapping. In these contexts, scalability across multi-core computing nodes becomes a key performance metric. In this section, we evaluate the parallel performance of KSW2, KSW2+Singletrack, WFA, BiWFA, and WFA+Singletrack when executed across multiple cores on our computing node.

To mitigate the effects of load imbalance, which are not the focus of this study and specifically affect the PromethION dataset, each core independently aligns the entire dataset. Execution time is calculated as the average runtime across all threads and normalized by dividing by the number of threads, yielding the scaled time. To maintain a consistent system load, once a core completes its alignment, it immediately begins a new alignment of the dataset if other cores are still processing their first alignment. This process continues until every core has completed at least one full alignment. Only the execution time for the first alignment on each core is included in the analysis. Multi-threaded speedup is then computed relative to single-threaded BiWFA performance by dividing the single-thread BiWFA execution time (reported in the previous section) by the scaled time observed for each algorithm using *N* threads. BiWFA serves as the baseline for speedup calculations because it consistently completes all test cases without exhausting memory, whereas KSW2, KSW2+Singletrack, WFA, and WFA+Singletrack run out of memory in certain scenarios.

**Figure 3:**
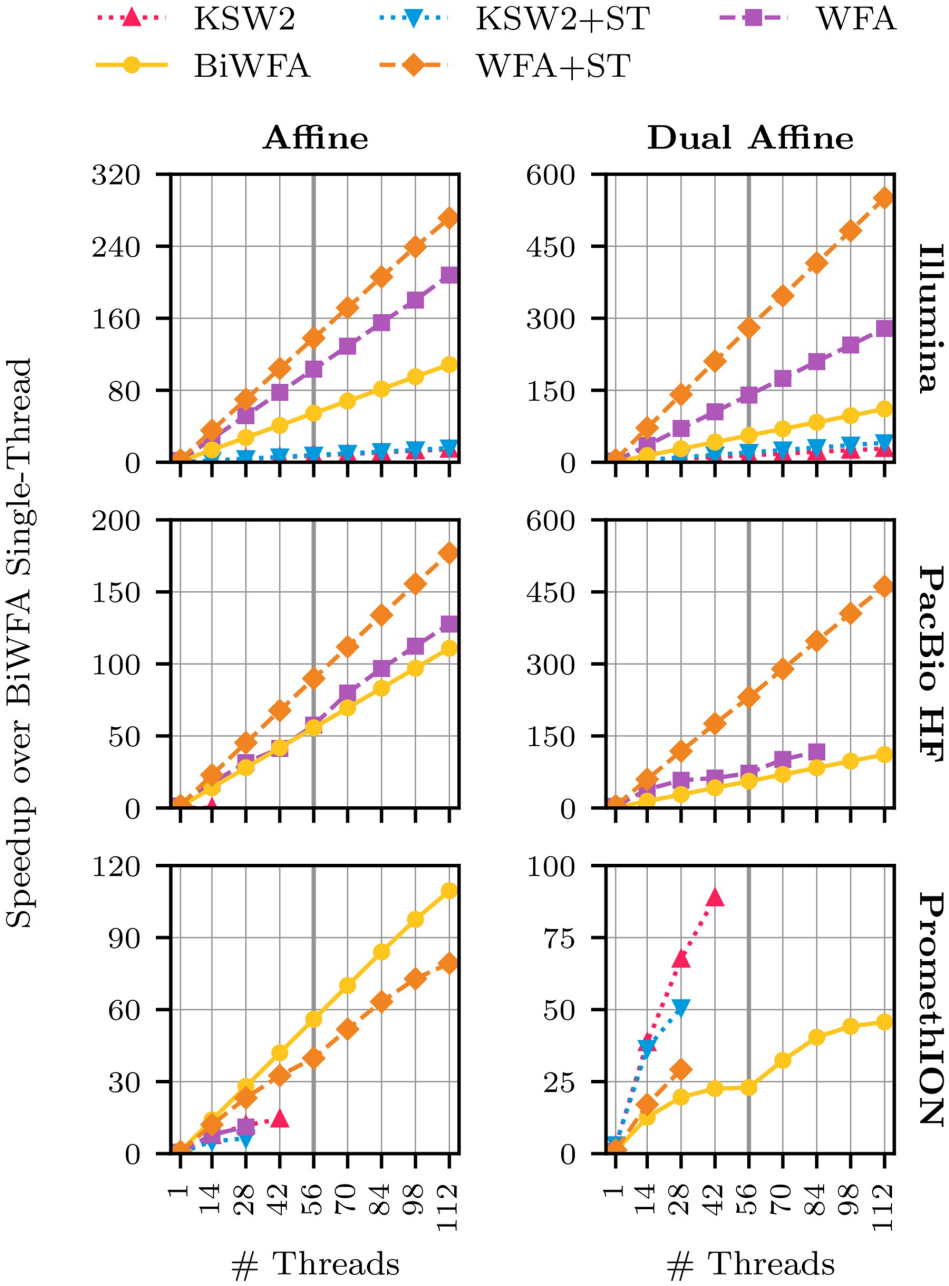
Multi-threaded speedups of KSW2, KSW2+ST, WFA, BiWFA, and WFA+ST relative to single-threaded BiWFA (ST = Singletrack).

Figure 3 shows the speedup of each algorithm relative to BiWFA single-threaded execution for thread counts of 1, 14, 28, 42, 56, 70, 84, 98, and 112. In this figure, rows correspond to datasets, and columns to cost functions. That is, each subplot corresponds to a dataset-function pair. Note that the y-axis range varies across subplots. The computing node consists of two chips: thread counts up to 56 utilise a single chip, while higher thread counts engage both chips. This architectural transition is indicated by a thick vertical line at 56 threads. In some cases, the speedup curves end before reaching 112 threads because the algorithm exhausts the node’s available memory. All algorithms, however, always find the optimal alignment as long as they do not run out of memory.

Since each core performs the same workload as in the single-threaded benchmarks, total memory usage at a given thread count can be precisely computed by multiplying the per-thread memory reported in Table 2 by the number of threads. The resulting multi-threaded memory usage is provided in the Supplementary Material, available as supplementary data at *Bioinformatics* online.

The parallel performance of each algorithm is influenced by the use of shared resources, such as the last-level cache (LLC) and main memory. When alignment tasks fit entirely within the private L1 and L2 caches of each core, the algorithms achieve ideal speedups: the scaled execution time with *N* threads matches that of single-threaded execution. This is the case for all algorithms on the Illumina dataset, which comprises short sequences.

As memory requirements increase, particularly with the dual gap-affine cost function, WFA, KSW2, and KSW2+Singletrack increasingly rely on shared resources, causing deviations from ideal performance. In contrast, both BiWFA and WFA+Singletrack maintain ideal or near-ideal scaling in most cases due to their more efficient utilization of the memory hierarchy. Consequently, and consistent with the single-threaded performance results discussed previously, WFA+Singletrack outperforms both BiWFA and WFA in the majority of executions in which it does not exhaust the available memory. Notably, WFA+Singletrack’s efficient memory usage enables higher thread counts on large datasets than WFA. For example, when aligning the PacBio HiFi dataset using the dual-gap affine scoring, WFA+Singletrack can utilize all 112 cores on the node, whereas WFA exhausts available memory beyond 84 threads.

KSW2+Singletrack achieves better parallel performance than KSW2 for the Illumina experiments in both gap-affine and dual gap-affine alignments, as well as for the PacBio HiFi experiments under dual gap-affine (before exhausting memory), consistent with the single-threaded results. It also outperforms KSW2 in the PromethION experiments under dual gap-affine at low thread counts. In contrast, KSW2 shows better parallel performance than KSW2+Singletrack for the PacBio HiFi and PromethION datasets under gap-affine and for the PromethION dataset under dual gap-affine, across 14–42 threads. Notably, either KSW2 or KSW2+Singletrack is the best-performing algorithm for PromethION under dual gap-affine before reaching the node’s memory limit. While KSW2’s lower memory requirements allow it to reach higher thread counts than KSW2+Singletrack, the memory demands of both algorithms ultimately constrain their scalability, leading to memory exhaustion at low thread counts for the PacBio and PromethION datasets.

For the largest datasets, the speedup curves display two distinct plateaus: the first occurs when the shared resources of a single chip are fully utilised (up to 56 cores), and the second occurs when the resources of both chips are fully utilised (beyond 56 cores). This behaviour is particularly evident for BiWFA with the dual gap-affine cost function on the PromethION dataset, where frequent access to shared resources constrains speedups.

At equivalent thread counts, WFA+Singletrack achieves up to a 3.2× speedup over WFA and a maximum speedup of 5.2× over BiWFA. Meanwhile, KSW2+Singletrack reaches speedups of up to 1.4× over KSW2 at low thread counts, while KSW2 exhibits better scalability and is thus preferable for fully loaded nodes. On the other hand, BiWFA shows better parallel performance than all other algorithms on the PromethION dataset under gap-affine scoring and can utilize all available cores in dual gap-affine mode. Consequently, algorithms implementing Singletrack are best suited for short to moderately large sequences.

## 7 Conclusion

In this work, we present Singletrack, a general backtrace algorithm that enables the computation of optimal gap-affine and dual gap-affine alignments with low memory requirements (comparable to gap-linear alignments), without adding alignment overheads, and with a simple, straightforward integration into existing tools.

We demonstrate that Singletrack reduces memory consumption in both the Suzuki-Kasahara (SK) and Wavefront Alignment (WFA) algorithms. For SK, memory usage is reduced by 2× for gap-affine and 4× for dual gap-affine alignments. For WFA, the reductions are 3× and 5×, respectively.

We implement Singletrack in the KSW2 and WFA2-lib libraries. Our results show that Singletrack provides performance improvements during the alignment step. In KSW2, replacing the backtrace matrix memory-reduction approach with Singletrack results in up to 1.4× speedups, at the cost of roughly doubling memory usage. In WFA2-lib, Singletrack provides speedups of 1.2–2.1×. We also compared our Singletrack-enhanced WFA to BiWFA (also included in WFA2-lib), which employs a divide-and-conquer approach to achieve linear memory usage. Our experiments show that WFA with Singletrack achieves up to 5.2× higher performance than BiWFA, at the cost of increased memory consumption, making it a compelling option for aligning moderately large sequences.

In summary, our method offers a practical solution to overcome the memory limitations of gap-affine and dual gap-affine sequence alignment, while also providing additional performance improvements during alignment. Moreover, Singletrack’s lightweight implementation integrates easily with existing alignment tools, enhancing their practicality and availability for the bioinformatics community. Notably, the Singletrack backtrace has already been adopted in Theseus, extending its applicability to sequence-to-graph alignment Jiménez-Blanco et al. (2026). We expect Singletrack to contribute to the development of more efficient and scalable bioinformatics tools that can handle large-scale sequencing datasets in the future.

## Supplementary Material

btag183_Supplementary_Data

## Data Availability

The datasets used for the experimental evaluation in this article are available in Zenodo at https://doi.org/10.5281/zenodo.17525721.
