# Supplementary material for: Singletrack: an algorithm for improving memory consumption and performance of gap-affine sequence alignment
Source: Bioinformatics. 2026 Apr 13;42(5):btag183. doi: 10.1093/bioinformatics/btag183 (PMC13171181; doi:10.1093/bioinformatics/btag183)
Supplement: btag183_Supplementary_Data [file btag183_supplementary_data.pdf]

Singletrack: An Algorithm for Improving Memory Consumption and Performance of Gap-Affine Sequence Alignment

Supplementary Material

Lorién López-Villellas[1], Cristian Iñiguez[2], Albert Jiménez-Blanco[2], Quim Aguado-Puig[4], Miquel Moretó[3,2], Jesús Alastruey-Benedé[1], Pablo Ibáñez[1], and Santiago Marco-Sola[3,2]

[1]Departamento de Informática e Ingeniería de Sistemas / Aragón Institute for Engineering Research (I3A), Universidad de Zaragoza, Spain
[2]Barcelona Supercomputing, Spain
[3]Department of Computer Science, Universitat Politècnica de Catalunya, Spain
[4]Departament d'Arquitectura de Computadors i Sistemes Operatius, Universitat Autònoma de Barcelona, Spain

Summary

This supplementary material provides additional details and technical content that complement the main manuscript, including:

- Extended classical gap-affine and dual gap-affine equations.
- The classical backtrace algorithm for gap-affine alignment.
- Dual gap-affine recurrence relations in the Suzuki-Kasahara formulation.
- Dual gap-affine recurrence relations for the Wavefront Alignment Algorithm (WFA).
- The Singletrack backtrace algorithm for dual gap-affine alignment.
- A detailed description of the Singletrack backtrace algorithm applied to the WFA.
- Command lines for reproducing the experiments in the main paper.
- Multi-threaded memory usage of the evaluated algorithms.

1 Gap-Affine and Dual Gap-Affine Extended Equations

Equations 1 and 2 present the complete recurrence relations, including the computation of boundary cells, for the DP matrices used in the gap-affine and dual gap-affine scoring functions, respectively.

$$
\begin{aligned}
I1_{i,j} &= \begin{cases} \infty & (i \geq 0, j = 0) \\ o_1 + j \cdot e_1 & (i = 0, j > 0) \\ \min \begin{cases} I1_{i,j-1} + e_1 \\ M_{i,j-1} + o_1 + e_1 \end{cases} & (i > 0, j > 0) \end{cases} \\
D1_{i,j} &= \begin{cases} o_1 + i \cdot e_1 & (i > 0, j = 0) \\ \infty & (i = 0, j \geq 0) \\ \min \begin{cases} D1_{i-1,j} + e_1 \\ M_{i-1,j} + o_1 + e_1 \} \end{cases} & (i > 0, j > 0) \end{cases} \\
M_{i,j} &= \begin{cases} 0 & (i = 0, j = 0) \\ o_1 + i \cdot e_1 & (i > 0, j = 0) \\ o_1 + j \cdot e_1 & (i = 0, j > 0) \\ \min \begin{cases} I1_{i,j} \\ D1_{i,j} \\ M_{i-1,j-1} + S(i,j) \end{cases} & (i > 0, j > 0) \end{cases}
\end{aligned}
\tag{1}
$$

$$
\begin{aligned}
I1_{i,j} &= \begin{cases} \infty & (i \geq 0, j = 0) \\ o_1 + j \cdot e_1 & (i = 0, j > 0) \\ \min \begin{cases} I1_{i,j-1} + e_1 \\ M_{i,j-1} + o_1 + e_1 \end{cases} & (i > 0, j > 0) \end{cases} \\
D1_{i,j} &= \begin{cases} o_1 + i \cdot e_1 & (i > 0, j = 0) \\ \infty & (i = 0, j \geq 0) \\ \min \begin{cases} D1_{i-1,j} + e_1 \\ M_{i-1,j} + o_1 + e_1 \end{cases} & (i > 0, j > 0) \end{cases} \\
I2_{i,j} &= \begin{cases} \infty & (i \geq 0, j = 0) \\ o_2 + j \cdot e_2 & (i = 0, j > 0) \\ \min \begin{cases} I2_{i,j-1} + e_2 \\ M_{i,j-1} + o_2 + e_2 \end{cases} & (i > 0, j > 0) \end{cases} \\
D2_{i,j} &= \begin{cases} o_2 + i \cdot e_2 & (i > 0, j = 0) \\ \infty & (i = 0, j \geq 0) \\ \min \begin{cases} D2_{i-1,j} + e_2 \\ M_{i-1,j} + o_2 + e_2 \end{cases} & (i > 0, j > 0) \end{cases} \\
M_{i,j} &= \begin{cases} 0 & (i = 0, j = 0) \\ \min \begin{cases} o_1 + i \cdot e_1 \\ o_2 + i \cdot e_2 \end{cases} & (i > 0, j = 0) \\ \min \begin{cases} o_1 + j \cdot e_1 \\ o_2 + j \cdot e_2 \end{cases} & (i = 0, j > 0) \\ \min \begin{cases} I1_{i,j} \\ D1_{i,j} \\ I2_{i,j} \\ D2_{i,j} \\ M_{i-1,j-1} + S(i,j) \end{cases} & (i > 0, j > 0) \end{cases}
\end{aligned}
\tag{2}
$$

2 Classical Backtrace Algorithm

Algorithm 1 presents the classical backtrace algorithm for the gap-affine model, which requires access to all three DP matrices: M, $I1$, and $D1$.

Algorithm 1 Classical gap-affine backtrace

Input Sequences $q_{0,\ldots,n-1}$, $t_{0,\ldots,m-1}$, matrices $M, I1, D1$, penalties $\{a, x, o_1, e_1\}$

Output CIGAR string that represents the optimal alignment

```
function BACKTRACE(q, t, M, I1, D1, p = {a, x, o_1, e_1})
    Definition: PUSH_OP(cigar, op, len) appends op len
    times to cigar.
    cigar ← ε, i ← n, j ← m, state ← M
    while i > 0 or j > 0 do
        if state = M then
            if M_{i,j} = I_{i,j} then
                state ← I
            else if M_{i,j} = D_{i,j} then
                state ← D
            else
                if q_{i-1} = t_{j-1} then
                    PUSH_OP(cigar, ‘M’, 1)
                else
                    PUSH_OP(cigar, ‘X’, 1)
                end if
                i ← i − 1, j ← j − 1
            end if
        else if state = I and j > 0 then
            if I_{i,j} ≠ I_{i,j-1} + e_1 then
                state ← M
            end if
            PUSH_OP(cigar, ‘I’, 1)
            j ← j − 1
        else if state = D and i > 0 then
            if D_{i,j} ≠ D_{i-1,j} + e_1 then
                state ← M
            end if
            PUSH_OP(cigar, ‘M’, 1)
            i ← i − 1
        else
            state ← M
        end if
    end while
    return cigar
end function
```

3 Dual Gap-Affine Equations in Suzuki-Kasahara Formulation

Equation 3 shows the recurrence relations for the dual gap-affine scoring function in the Suzuki-Kasahara formulation. Note that the A matrix is not stored; it is only used as a temporary variable to compute ΔH and ΔV.

$$
\begin{aligned}
A_{i,j} &= \min \begin{cases} S(i,j) \\ \Delta E1_{i,j-1} + \Delta V_{i,j-1} + e_1 \\ \Delta F1_{i-1,j} + \Delta H_{i-1,j} + e_1 \\ \Delta E2_{i,j-1} + \Delta V_{i,j-1} + e_2 \\ \Delta F2_{i-1,j} + \Delta H_{i-1,j} + e_2 \end{cases} \\
\Delta H_{i,j} &= A_{i,j} - \Delta V_{i,j-1} \\
\Delta V_{i,j} &= A_{i,j} - \Delta H_{i-1,j} \\
\Delta E1_{i,j} &= \min \begin{cases} o_1 \\ \Delta E1_{i,j-1} - \Delta H_{i,j} + e_1 \end{cases} \\
\Delta F1_{i,j} &= \min \begin{cases} o_1 \\ \Delta F1_{i-1,j} - \Delta V_{i,j} + e_1 \end{cases} \\
\Delta E2_{i,j} &= \min \begin{cases} o_2 \\ \Delta E2_{i,j-1} - \Delta H_{i,j} + e_2 \end{cases} \\
\Delta F2_{i,j} &= \min \begin{cases} o_2 \\ \Delta F2_{i-1,j} - \Delta V_{i,j} + e_2 \end{cases}
\end{aligned}
\tag{3}
$$

4 Dual Gap-Affine Equations in Wavefront Alignment Algorithm

Equation 4 shows the recurrence relations for the dual gap-affine scoring function in WFA.

$$
\begin{aligned}
\widetilde{I1}_{z,k} &= \max \begin{cases} \widetilde{M}_{z-o_1-e_1,k-1} \\ \widetilde{I1}_{z-e_1,k-1} + 1 \end{cases} \\
\widetilde{D1}_{z,k} &= \max \begin{cases} \widetilde{M}_{z-o_1-e_1,k+1} \\ \widetilde{D1}_{z-e_1,k+1} \end{cases} \\
\widetilde{I2}_{z,k} &= \max \begin{cases} \widetilde{M}_{z-o_2-e_2,k-1} \\ \widetilde{I2}_{z-e_2,k-1} + 1 \end{cases} \\
\widetilde{D2}_{z,k} &= \max \begin{cases} \widetilde{M}_{z-o_2-e_2,k+1} \\ \widetilde{D1}_{z-e_2,k+1} \end{cases} \\
\widetilde{M}_{z,k} &= \max \begin{cases} \widetilde{M}_{z-x,k} + 1 \\ \widetilde{I1}_{z,k} \\ \widetilde{D1}_{z,k} \\ \widetilde{I2}_{z,k} \\ \widetilde{D2}_{z,k} \end{cases}
\end{aligned}
\tag{4}
$$

5 Singletrack Dual Gap-Affine Backtrace

Algorithm 2 presents the pseudocode of the Singletrack backtrace algorithm for the dual gap-affine cost function.

Algorithm 2 Singletrack dual gap-affine backtrace

Input Sequences $q_{0,\ldots,n-1}$, $t_{0,\ldots,m-1}$, matrices $M, I1, D1, I2, D2$, penalties $\{a, x, o_1, e_1, o_2, e_2\}$

Output CIGAR string that represents the optimal alignment

function BACKTRACE($q, t, M, p = \{a, x, o_1, e_1, o_2, e_2\}$)
  Definition: `PUSH_OP(cigar, op, len)` appends `op` `len` times to `cigar`.
  $\text{cigar} \leftarrow \epsilon, i \leftarrow n, j \leftarrow m, \text{state} \leftarrow M$
  while $i > 0$ **or** $j > 0$ **do**
    if $\text{state} = M$ **then**
      if $i > 0$ **and** $j > 0$ **and** $M_{i,j} = M_{i-1,j-1} + S(i,j)$ **then**
        if $q_{i-1} = t_{j-1}$ **then**
          PUSH_OP(cigar, 'M', 1)
        else
          PUSH_OP(cigar, 'X', 1)
        end if
        $i \leftarrow i - 1,\ j \leftarrow j - 1$
      else
        $l \leftarrow 0, \text{state} \leftarrow \bar{M}$
      end if
    else
      $l \leftarrow l + 1$
      $s'_1 \leftarrow M_{i,j} - o_1 - l \cdot e_1$
      $s'_2 \leftarrow M_{i,j} - o_2 - l \cdot e_2$
      if $j - l \geq 0$ **and** $s'_1 = M_{i,j-l}$ **then**
        PUSH_OP(cigar, 'I', l)
        $j \leftarrow j - l, \text{state} \leftarrow M$
      else if $i - l \geq 0$ **and** $s'_1 = M_{i-l,j}$ **then**
        PUSH_OP(cigar, 'D', l)
        $i \leftarrow i - l, \text{state} \leftarrow M$
      else if $j - l \geq 0$ **and** $s'_2 = M_{i,j-l}$ **then**
        PUSH_OP(cigar, 'I', l)
        $j \leftarrow j - l, \text{state} \leftarrow M$
      else if $i - l \geq 0$ **and** $s'_2 = M_{i-l,j}$ **then**
        PUSH_OP(cigar, 'D', l)
        $i \leftarrow i - l, \text{state} \leftarrow M$
      end if
    end if
  end while
  return cigar
end function

6 Extended Description of Singletrack in Wavefront Alignment Algorithm

In the main text, we used the Wavefront Alignment Algorithm [1] (WFA) as a case study to evaluate the impact of the Singletrack backtrace algorithm. Here, we provide specific details for integrating Singletrack into WFA. We focus on the gap-affine scoring model, but the approach generalizes seamlessly to the dual gap-affine score function.

Analogous to the general Singletrack strategy, we assume that the WFA backtrace algorithm only has access to the $\widetilde{M}_{0,\ldots,s}$ wavefronts, where s denotes the optimal alignment score. It is important to note that WFA does not explicitly store all computed DP cells due to its use of the `extend` operation. After generating a DP cell with score z, diagonal k, and offset off' using the `next` operation, the `extend` operation increases the offset as long as matches are found along the diagonal, ultimately storing only the offset off corresponding to the furthest-reaching cell for each diagonal and score. Consequently, when considering a current cell $off = \widetilde{M}_{z,k}$, the original offset off' before extension is not directly available. To address this, the Singletrack algorithm performs a reverse extension (`rev_extend`) operation. Starting from the current cell $off = \widetilde{M}_{z,k}$, `rev_extend` decrements the offset while matches are found along the diagonal in reverse, recovering the tentative original offset off'. Once this reverse extension is complete, we determine the origin of the predecessor cell by checking if off' is consistent with the expected predecessor in the M matrix, specifically whether off' equals $\widetilde{M}_{z-x,k} + 1$ for a substitution. If this condition is satisfied, the predecessor cell is in the M matrix (i.e., in a $\widetilde{M}$ wavefront), and the algorithm continues the backtrace from this position. If the condition is not true, the predecessor must be in one of the indel wavefronts, either $\widetilde{I}1$ or $\widetilde{D}1$. At this stage, we open two tentative backtrace paths: one assuming the current cell originated from $\widetilde{I}1$, and the other from $\widetilde{D}1$. For these tentative paths, the offset off' obtained from `rev_extend` may not correspond to the original offset of $\widetilde{M}z - o_1 - e_1, k$. The real original offset could be any value between off' and off, since the insertion or deletion may have occurred in the middle of a chain of consecutive matches along the diagonal. Knowing this, the algorithm increases the gap length l iteratively, computing the expected score z' and checking for a consistent path back to M. In the case of insertions, this is done by verifying if off' is less than or equal to $\widetilde{M}_{z',k-l} + l$ and less than or equal to off. For deletions, the check is if off' is less than or equal to $\widetilde{M}_{z',k+l}$ and less than or equal to off. When one of these criteria is satisfied, the corresponding path is confirmed as the correct backtrace path, and the other path is discarded. The algorithm then resumes the backtrace from the identified M cell. The pseudocode for the Singletrack backtrace algorithm in WFA is presented in Algorithm 3.

We reuse the example from the main text to demonstrate the Singletrack backtrace in WFA. Figure 1 shows the DP cells and wavefronts computed by WFA for the alignment of sequences $q =$ `GCA` and $t =$ `GCCAA`. The $\widetilde{I}1$ and $\widetilde{D}1$ wavefronts are shown to provide full context for the example, although they are never used by the Singletrack backtrace algorithm itself. The alignment begins at cell $M_{3,5}$ (the current cell), which corresponds to $\widetilde{M}_{10,2} = 5$. Here, $z = s = 10$, and the diagonal and offset are $k = 2$ and $off = 5$, respectively. Since we start in M, the Singletrack algorithm performs the `rev_extend` operation. In this case, there is one match (the letter `A`) on diagonal $k = 2$ starting at $off = 5$, so $off' = 4$. However, the cell preceding $\widetilde{M}_{10,2}$ before executing the `extend` operation was not generated from a substitution, since $off' \neq \widetilde{M}_{z',2}+1$, with $z' = z-x = 6$.

Algorithm 3 Gap-affine Singletrack backtrace in WFA

Input Sequences $q_{0,\dots,n-1}$, $t_{0,\dots,m-1}$, optimal score s, list of wavefronts $\widetilde{M}_{0,\dots,s}$, penalties $\{a = 0, x > 0, o_1 > 0, e_1 > 0\}$

Output CIGAR string that represents the optimal alignment

function BACKTRACE($q, t, s, \widetilde{M}_{0,\dots,s}, p = \{a = 0, x > 0, o_1 > 0, e_1 > 0\}$)
  Definition: `PUSH_OP(cigar, op, p)` adds `op` to `cigar` `p` times.
  Definition: $S(i, j) = a$ if $q_{i-1} = t_{j-1}$, x otherwise.
  ▷ For simplicity, we assume that an invalid access to $\widetilde{M}_{0,\dots,s}$ (either invalid score or diagonal) returns $-\infty$
  $\text{cigar} \leftarrow \epsilon, z \leftarrow s, l \leftarrow 0, k \leftarrow m - n, off \leftarrow m, off' \leftarrow 0, \text{state} \leftarrow M$
  while $s > 0$ **do**
    if state $= M$ **then**
      $i \leftarrow off - k, j \leftarrow off, off' \leftarrow off$
      while $i > 0$ **and** $j > 0$ **and** $q_{i-1} = t_{j-1}$ **do** ▷ Reverse extend()
        $i \leftarrow i - 1, j \leftarrow j - 1, off' \leftarrow off' - 1$
      end while
      $z' \leftarrow z - x$
      if $\widetilde{M}_{z',k} + 1 = off'$ **then**
        PUSH_OP(cigar, 'M', $off' - off$)
        PUSH_OP(cigar, 'X', 1)
        $z \leftarrow z', off = off'$
      else
        $l \leftarrow 0, \text{state} \leftarrow \bar{M}$
      end if
    else
      $l \leftarrow l + 1, z' \leftarrow z - o_1 - l \cdot e_1$
      if $off' \leq \widetilde{M}_{z',k-l} + l \leq off$ **then**
        PUSH_OP(cigar, 'M', $off - (\widetilde{M}_{z',k-l} + l)$)
        PUSH_OP(cigar, 'I', l)
        $off \leftarrow \widetilde{M}_{z',k-l}, k \leftarrow k - l, z \leftarrow z', \text{state} \leftarrow M$
      else if $off' \leq \widetilde{M}_{z',k+l}\widetilde{M}_{z',k+l} \leq off$ **then**
        PUSH_OP(cigar, 'M', $off - \widetilde{M}_{z',k+l}$)
        PUSH_OP(cigar, 'I', l)
        $off \leftarrow \widetilde{M}_{z',k+l}, k \leftarrow k + l, z \leftarrow z', \text{state} \leftarrow M$
      end if
    end if
  end while
  PUSH_OP(cigar, 'M', off)
  return cigar
end function

Therefore, the predecessor cell must be in either $I1$ or $D1$. At this point, the gap length l is initialized to 0 and then increased to 1, and two tentative paths have been opened. The following conditions are then checked: $off' \leq \widetilde{M}_{z',k-l}+l \leq off$ for the insertion path, and $off' \leq \widetilde{M}_{z',k+l} \leq off$ for the deletion path, where $z' = z - o_1 - l \cdot e_1 = 10 - 6 - 1 \cdot 2 = 2$. None of the conditions is true, so the gap length l is increased to 2. In this case, the condition $off' \leq \widetilde{M}_{z',k-l} + l \leq off$, where $z' = z - o_1 - l \cdot e_1 = 10 - 6 - 2 \cdot 2 = 0$ is satisfied, indicating that the optimal path to $M_{3,5}$ goes through $M_{2,2}$, $I1_{2,3}$, and $M_{2,4}$. At this stage, a match and two insertions are added to the CIGAR string, which is initialized as `IIM`. Upon reaching cell $M_{2,2}$ with score $z = 0$, the condition of the `while` loop is no longer satisfied, and the loop exits. Since $off > 0$, there remain off matches to be added to the CIGAR string, which are appended before completing the backtrace process. The final CIGAR string is thus `MMIIM`.

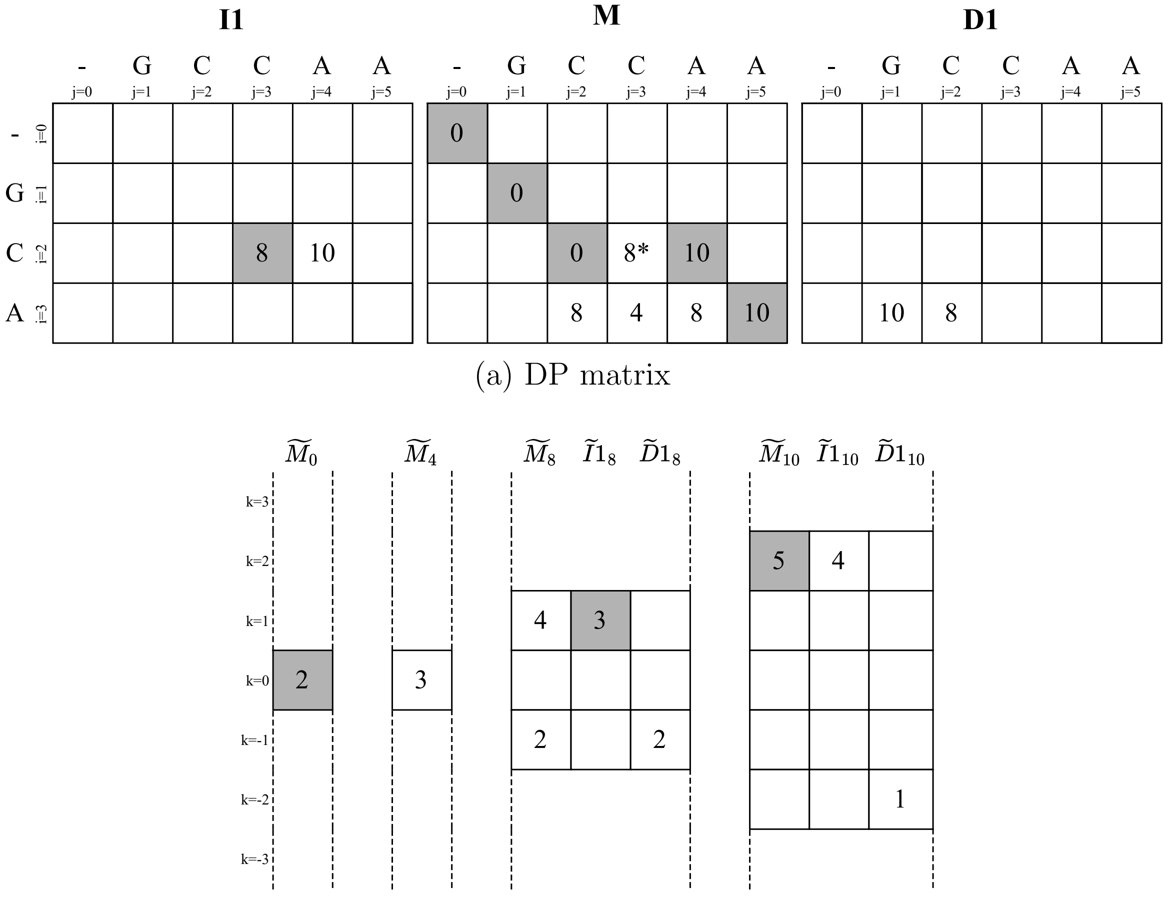

Figure 1: Computed cells of the DP matrix (a) and wavefronts (b) generated by WFA during the alignment of sequences q = `GCA` and t = `GCCAA` using the gap-affine cost function with penalties $a = 0$, $x = 4$, $o_1 = 6$, and $e_1 = 2$. DP matrix cells marked with an asterisk (*) indicate cells that are computed but not stored due to the `extend` operation. Empty cells in the wavefronts represent non-existent DP cells; that is, for a given score z and diagonal k, no DP cell exists with that score on that diagonal. Note that only a subset of the $\widetilde{I}1$ and $\widetilde{D}1$ wavefronts needs to be stored during alignment, as the Singletrack backtrace algorithm requires only the $\widetilde{M}$ wavefronts. Dark-shaded cells indicate matrix entries that are part of the optimal alignment path.

7 Command-Line Invocation of Alignment Tools

This section reports the exact command lines used to run each alignment tool in our experimental evaluation. All experiments perform global alignments and process a dataset file, denoted as `<dataset>`, containing all sequence pairs to be aligned.

The modified versions of KSW2 and WFA used in our experiments can be found on Zenodo (DOI:10.5281/zenodo.18770585) and GitHub (`https://github.com/LorienLV/singletrack`).

The paths `singletrack/ksw2_singletrack` refer to our modified version of KSW2 with support for Singletrack backtrace. This version can be executed either with the original backtrace (by omitting the `--single-track` flag) or with the Singletrack backtrace (by explicitly providing the `--single-track` flag). KSW2 and KSW2+Singletrack use a gap-affine scoring model when the flags `--match`, `--mismatch`, `--gapo`, and `--gape` are specified, and a dual gap-affine model when these are combined with `--gapo2` and `--gape2`.

The path `WFA2-lib` corresponds to the original, unmodified WFA2-lib, whereas `singletrack/WFA2-lib` denotes our version modified to use Singletrack for backtracing. The scoring function is selected using the `-a` flag, with `gap-affine-wfa` for gap-affine scoring and `gap-affine2p-wfa` for dual gap-affine scoring. In all cases, the `--wfa-span` flag is set to `global`, as only global alignments are considered in our experiments.

The following command lines were used.

- **KSW2**

```
/usr/bin/time -v \
singletrack/ksw2_singletrack/benchmark_singletrack \
-d <dataset> \
--match 0 --mismatch 4 --gapo 6 --gape 2 \
[--gapo2 24 --gape2 1]
```

- **KSW2+Singletrack**

```
/usr/bin/time -v \
singletrack/ksw2_singletrack/benchmark_singletrack \
--single-track -d <dataset> \
--match 0 --mismatch 4 --gapo 6 --gape 2 \
[--gapo2 24 --gape2 1]
```

- **WFA**

```
/usr/bin/time -v \
WFA2-lib/build/align_benchmark --wfa-memory high \
-a {gap-affine-wfa|gap-affine2p-wfa} \
-i <dataset> --wfa-span global
```

- **BiWFA**

```
/usr/bin/time -v \
singletrack/WFA2-lib/build/align_benchmark \
--wfa-memory high -a {gap-affine-wfa|gap-affine2p-wfa} \
-i <dataset> --wfa-span global
```

- **WFA+Singletrack**

```
/usr/bin/time -v \
WFA2-lib/build/align_benchmark --wfa-memory ultralow \
-a {gap-affine-wfa|gap-affine2p-wfa} \
-i <dataset> --wfa-span global
```

8 Complementary Multi-Threaded Results

Figure 2 shows the multi-threaded memory consumption of the evaluated algorithms across varying thread counts. Memory usage scales linearly with the number of threads, since each thread performs the same set of alignments.

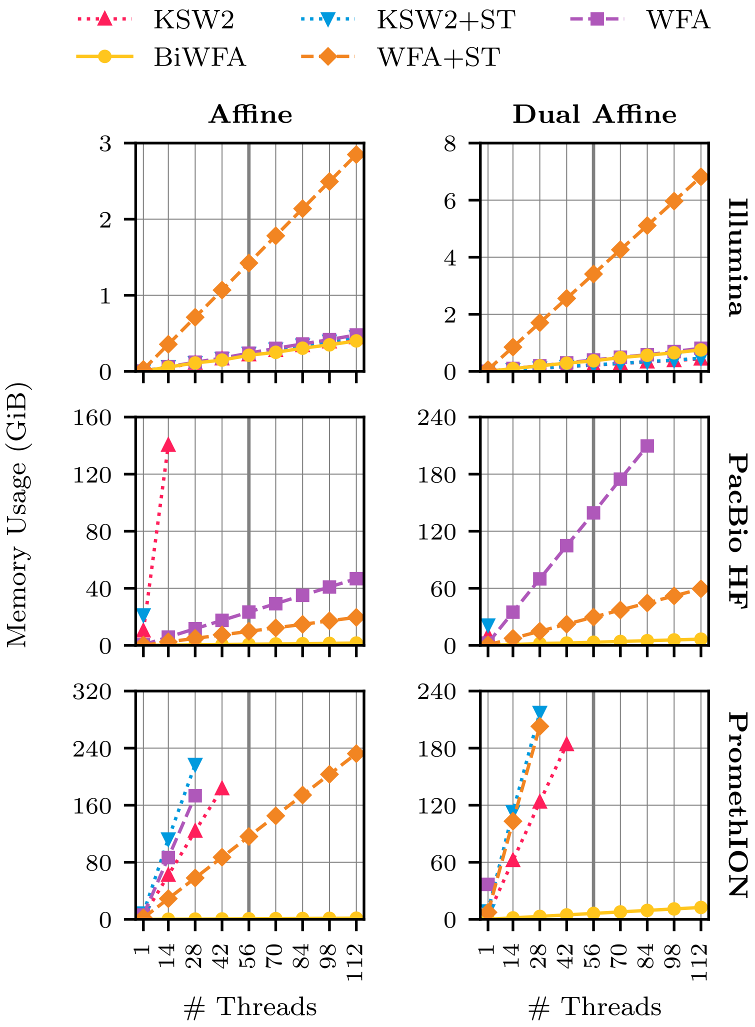

Figure 2: Multi-threaded memory usage in GiB of KSW2, KSW2+ST, WFA, BiWFA, and WFA+ST (ST = Singletrack).

References

[1] Santiago Marco-Sola, Juan Carlos Moure, Miquel Moreto, and Antonio Espinosa. Fast gap-affine pairwise alignment using the wavefront algorithm. *Bioinformatics*, 37(4):456–463, 09 2020.
